# A novel mutation in *SETX* and *ATM* causes ataxia in consanguineous Pakistani families

**DOI:** 10.12669/pjms.40.8.9246

**Published:** 2024-09

**Authors:** Rabia Akram, Shahid Mahmood Baig, Haseeb Anwar, Ghulam Hussain

**Affiliations:** 1 Rabia Akram, M Phil. Department of Physiology, Neurochemicalbiology and Genetics Laboratory (NGL), Faculty of Life Sciences, Government College University, Faisalabad, Pakistan; 2 Shahid Mahmood Baig, PhD. Human Molecular Genetics Laboratory, Health Biotechnology Division, National Institute for Biotechnology and Genetic Engineering (NIBGE) College, Faisalabad, Pakistan. Department of Biological and Biomedical Sciences, Aga Khan University, Karachi, Pakistan; 3 Haseeb Anwar, PhD. Department of Physiology, Neurochemicalbiology and Genetics Laboratory (NGL), Faculty of Life Sciences, Government College University, Faisalabad, Pakistan; 4 Ghulam Hussain, PhD. Department of Physiology, Neurochemicalbiology and Genetics Laboratory (NGL), Faculty of Life Sciences, Government College University, Faisalabad, Pakistan

**Keywords:** Consanguinity, Movement disorders, *SETX*, Spinocerebellar ataxia, Spastic paraplegia, Pakistan

## Abstract

**Background & Objectives:**

Ataxia is usually caused by cerebellar pathology or a decrease in vestibular or proprioceptive afferent input to the cerebellum. It is characterized by uncoordinated walking, truncal instability, body or head tremors, uncontrolled coordination of the hands, dysarthria, and aberrant eye movements. The objective of the current investigation was to identify the underlying genetic cause of the hereditary ataxia that affects the Pakistani population.

**Methods:**

We studied numerous consanguineous Pakistani families whose members had ataxia-related clinical symptoms to varying degrees. The families were chosen from the Punjab province, and the neurophysician conducted a clinical examination. Peripheral blood samples from both sick and healthy members of the family were taken after obtaining informed consent. Genomic DNA was used to find potential variations in probands using whole exome sequencing. The study was carried out at the University Hospital of Tübingen, Germany, and Government College University in Faisalabad, Pakistan, during 2018-2023.

**Results:**

The molecular analysis of these families identified different variants including *SGCB*: c.902C>T, c.668G>A, *ATM*: c.6196_6197insGAA, *SPG11*: c.5769del, *SETX* c.5525_5533del, and *ATM*: c.7969A>T. A noteworthy mutation in *ATM* and *SETX* was observed among them, and its symptoms were shown to cause ataxia in these families.

**Conclusion:**

The current study broadens the mutation spectrum of several hereditary ataxia types and suggests the next generation sequencing in conjunction with clinical research for a more accurate diagnosis of overlapping phenotypes of this disorder in the Pakistani population.

## INTRODUCTION

Movement disorders are a diverse collection of neurologic conditions marked by irregularities in tone, and posture, the inability to initiate or control voluntary movements, and the occurrence of unintentional movements.^1^ These problems may be inherited or because of different types of Central Nervous System (CNS) injury, or structural and functional changes to the basal ganglia circuit and other cerebral regions. Patients frequently have a combination of movement disorders when they first appear, in which cases identifying the primary movement disorder can be very helpful in developing a differential diagnosis.^2^

A group of movement disorders known as cerebellar ataxias are distinguished by the cerebellum’s gradual deterioration, which results in impairments of balance and gait.^3^ In this illness, signs of cerebellar degeneration are common, but they can also include pyramidal and extrapyramidal features, as well as other types of polyneuropathy. With an incidence of between 1.5 to 4.9 per 100,000 people, these neurodegenerative illnesses are characterized by the clinical condition of increasing incoordination brought on by cerebellar degeneration.^4^,^5^ Wide-based unstable gait, truncal instability, dysmetria, head or body tremors, dysdiadochokinesis, dyssynergia, uncontrolled hand coordination, dysarthria, and aberrant eye movements are the main clinical features of ataxia.^6^ These clinical findings led us to the discovery of five families with ataxia.

Overlapping presentations of phenotypes pose a significant obstacle in the differential diagnosis of these recessive cerebellar disorders^7^,^8^ Fortunately, accurate clinical phenotyping combined with next-generation sequencing (NGS) is a fundamental way to uncover the genetic variations driving these rare diverse disorders. This approach can also help us to understand the underlying pathomechanisms, which in turn can be used to develop better strategies for diagnosis and prognosis as well as more specialized and individualized treatments for these disorders. In this study, five consanguineous Pakistani families with the phenotype of ataxia and their related movement disorder were investigated using whole-exome sequencing (WES).^9^ We expanded and strengthened the genotypic-phenotypic spectrum of movement disorders by identifying novel as well as previously reported genetic variants in the consanguineous Pakistani population.

## METHODS

The research received approval from the Government College University Faisalabad (GCUF) Institutional Review Board (IRB-350; 01-06-2023) in Pakistan and the study was conducted during 2018-2023. It looked into five unrelated families with many affected members who had ataxia-related symptoms.

### Inclusion Criteria:

The families included in this study were recruited from the Punjab province of Pakistan. Expert neurophysicians evaluated patients by using different tests. After receiving informed consent, several elders were questioned about their family’s history of the illness. First and second-degree relatives with a history of unexplained gait abnormalities, speech difficulties, walking difficulties, developmental delay, cognitive dysfunction, and delayed milestones were required to meet the inclusion criteria.

### Exclusion Criteria:

- Sporadic cases included individuals with insufficient family histories or poor family history, the existence of recognized acquired ataxia,
- the ataxic syndrome linked to significant autonomic dysfunctions and/or atypical parkinsonism,
- disorders in which ataxia was a minor characteristic.^10^,^11^
- Moreover, conditions like alcoholism, vitamin deficiency, multiple sclerosis, vascular disease, primary or metastatic tumors, paraneoplastic diseases linked to occult carcinoma of the ovary, breast, or lung, and idiopathic degenerative disease multiple system atrophy (spinal muscular atrophy) were also excluded 12. These standards were created to make it possible to compare current research with prior studies and the more recent clinical-genetic categorization of ataxias in general.

### Sample collection:

EDTA-coated vacutainers were used to collect peripheral blood samples from both sick and healthy people, and they were kept at 4°C.

### Next generation sequencing:

Genomic DNA was isolated from peripheral blood samples. Probands V.3, V.3, IV.2, IV.2, and IV.1 from family A-E, respectively underwent WES utilizing an Illumina HiSeq 4000 sequencing machine and an Agilent (Santa Clara, CA) version six enrichment kit (paired-end reads, 2x75by) at University Hospital of Tübingen. The experiments and handling of the NGS data were done following the Moawia et al. The GRCh37 human genome assembly was used to align these sequencing reads. An internal VARBANK database and analytic tool kit were used to filter and prioritize the variants.^13^ A cut-off point for variations (single nucleotide variants/small indels) in the coding region and the surrounding intronic regions was set at minor allele frequency (MAF) 1.5 percent (68bp). The Human Genetic Variation database’s MAF cut-off value for known causative variables was up to 5% and up to 30 bp of flanking areas. The following databases were utilized to collect MAFs: dbSNP, 1000 Genomes, gnomAD, and an internal database. For more than 98 percent of the targeted locations, the coverage value was 30 high-quality sequencing reads per base.^14^

### In silico analyses of identified variants:

The GRCh37 genome assembly was utilized as a reference for variation mapping and annotation. Following that, the variants’ deleterious effects were evaluated using SIFT, Mutation taster, Polyphen-2 and CADD scores above 10. The American College of Medical Genetics and Genomics (ACMG) variant classification system was also used to highlight possibly pathogenic variants.^9^

## RESULTS

### Clinical findings:

**Fig.1:**
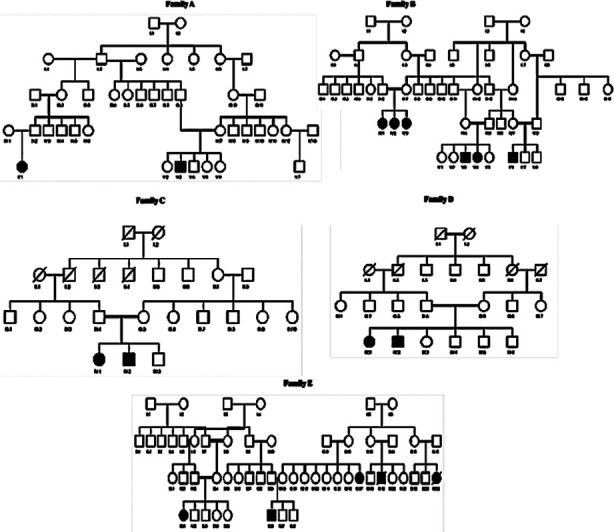
Pedigrees of families A through E showing how the disease runs within each family. A filled black box (male) and a circle (female) represent each of the affected people.

In this study, we examined five Pakistani families with distinctive neurological characteristics (Fig.1). A multiplex family, known as Family A, includes one sick person (V.3) and three healthy siblings who were born to consanguineous parents. Clinical signs displayed by the affected person included a gradual aberrant ataxic to no gait, stiffness, and muscle weakness. Family B has two affected individuals including a brother (V.3), and sister (V.4), and three unaffected siblings. Patient V.3 is a 12-year-old with a 2.5-year-old disease onset. Both people had gait abnormalities, difficulty walking, and muscle weakness as their clinical characteristics. Family C comprises two affected individuals, one male (IV.2) and one female (IV.1), and one healthy sibling from consanguineous parents. The individuals displayed ataxic gait, speech dysfunction, delayed milestones, muscle weakness, and walking difficulty at the age of 12. None of the patients showed signs of having a seizure.

Family D had two affected individuals (IV.1, IV.2). Individual IV.2’s condition began around the age of 22. Lower limb muscle weakness, strabismus, gait ataxia, dysarthria and walking difficulties were noted as symptoms. There were no impairments in intellect or hearing. Family E consisted of one affected individual (IV.1) with two healthy brothers and two healthy sisters. The disease began between the ages of 7 and 10, and it becomes worse with time. The patient exhibited an ataxic gait, muscle weakness, and a challenging gait.

### Genetic findings:

Genetic analysis of these families showed interesting results listed in Table-I.

**Table-I:** Variant Annotation, and Allele Frequencies of the Variants Identified in Family A–E.

		Family A	Family B	Family C	Family D	Family E

	Gene	SGCB	ATM	SPG11	SETX	ATM
Variant Annotation	Transcript	ENST00000381431	ENST00000278616	ENST00000261866	ENST00000224140	ENST00000278616
	GRCh37/hg38 position (DNA change)	4:52890178, 4:52894219	11:108186838	15:44876109	9:135176032	11:108204654
	cDNA change	c.902C>T, c.668G>A	c.6196_6197insGAA	c.5769del	c.5525_5533del	c.7969A>T
	Protein change	p.Thr301Ile, p.Arg223His	p.Gln2066delinsArgLys	p.Ser1923ArgfsTer28	p.His1842_Phe1844del	p.Lys2657Ter
	Variant type	Missense	Inframe insertion	Nonsense gain	Inframe 9bp deletion	Nonsense gain
	Zygosity	Het	Hom	Hom	Hom	Hom
Allele Frequencies	dbSNP ID	N/A	N/A	rs312262770	N/A	N/A
	genomAD2.1 (highest subpopulation)	0.0001	N/A	0.0001	N/A	N/A
	ACMG Classification	Uncertain significance	Uncertain significance	pathogenic	Uncertain significance	pathogenic

Hom: Homozygous; Het: Heterozygous; N/A: Not applicable.

## DISCUSSION

We describe the clinical picture seen in five unrelated families with muscular dystrophy (Family-A), ataxia-telangiectasia (Family B and E), spastic paraplegia 11 (Family-C), and spinocerebellar ataxia, autosomal recessive, with axonal neuropathy 2 (SCA2; Family D). We found novel mutations in *SGCB*: c.902C>T, *SETX*: c.5525_5533del, *ATM*: c.6196_6197insGAA and already reported mutations in *SPG11*: c.5769del gene. A mutation in *ATM:* c.7969A>T was also found in the *BRCA2* gene that is linked to breast carcinoma.

Family A revealed an interesting missense mutation in the *SGCB* gene at a position of exon 6. This heterozygous novel mutation (c.902C>T) is of uncertain significance and is found as the main cause of muscular dystrophy in this family as the clinical picture fits with this variant. The multisubunit protein complex known as sarcoglycan beta (*SGCB*; also known as the dystrophin-glycoprotein complex) spans the sarcolemma and provides structural connectivity between the extracellular matrix of muscle cells and the subsarcolemmal cytoskeleton.^15^ In an Iranian cohort, novel homozygous and heterozygous variants in the *SGCB* gene were discovered as the cause of limb muscular dystrophy.^16^–^18^

Different *ATM* gene variations were found in two distinct families. Family E has the pathogenic c.7969A>T, but family B has the variable c.6196 6197insGAA, which is of unknown significance (VUS). Ataxia-telangiectasia (A-T) is a neuroimmunological disorder brought on by mutated pathogenic variations of *ATM* gene. *ATM* gene encodes serine-threonine kinase which controls DNA repair and damage responses.^19^ Some cases related to this gene have been reported so far,^20^,^21^ however, Tariq et al. discovered a variation in exon 52 of *ATM* for the first time in the Pakistani ethnic group.^22^

A homozygous variation in *SPG11*: c.5769del (p.Ser1923Argfs*28) was found in Family C, and it revealed a deletion of 1 base pair in exon 30. A frameshift is produced by this variant beginning at codon Ser1923.^23^,^24^affecting 1-3% of the general population. Although research into the genetic causes of ID has recently gained momentum, identification of pathogenic mutations that cause autosomal recessive ID (ARID At position 28, the new reading frame ends with a STOP codon. It has the identifier rs312262770 in dbSNP and is recognized by ClinVar as RCV000034228. 2. The variant is listed in human polymorphism databases like genomAD. Spatacsin, a protein involved in the growth, and intracellular cargo trafficking of neuronal axons, is produced by the *SPG11* gene.^25^ This gene revealed that codon 247 experiences a frameshift and premature termination in the Italian siblings where ataxia and cognitive decline are the major signs of disease onset at the age of 12 and 15, respectively. However, the condition quickly worsened, resulting in dysarthria, spastic paraplegia, and peripheral neuropathy.^26^

In Family D, the *SETX* mutation c.5525 5533del was seen where the mode of inheritance was autosomal recessive (OMIM ID: 608465). Senataxin, a protein that is encoded by the *SETX* gene, possesses DNA/RNA helicase activity and is thought to be essential for DNA double-strand repair and RNA splicing pathways.^27^ Various studies help in diagnosis by adding the phenotypic spectrum of *SETX*-associated disorders.^28^–^30^ Interestingly, recent studies explored novel mutation 22 as well as already reported^9^ in the *SETX* gene in the Pakistani population, consequently improving the clinical manifestations and mutation spectrum, reinforcing the significance of assessing such cases in a Pakistani cohort going forward.

This study increases the clinical manifestations associated with *SGCB, SETX*, and *ATM-*related diseases and broadens their mutation range. This work is significant since it reports on new SCA2 phenotypes, identifies a new disease variation, and reveals the family’s unique ethnicity. Additionally, this might assist in identifying the families for cascade testing to lower the illness burden through prenatal genetic testing.

### Limitations of the Study:

The work is limited to the fact that genomic analyses offer strong evidence of the pathogenicity of the discovered variations. However, co-segregation and functional validation have not been carried out for the prediction of their precise pathomechanism.

## CONCLUSION

The current study broadens the mutation spectrum of movement disorders and reinforces the value of NGS as an effective and practical method for determining the underlying cause. The results indicate interesting mutations in *SETX* and *ATM* genes that may be the leading cause of ataxia. Therefore, our outlooks for the future include Sanger sequencing of other family members to validate the disease-causing variant. Apart from finding out the causative genes, this study will contribute to better genetic counseling and carrier screening options as well as an improvement in the diagnosis and prognosis of uncommon hereditary ataxia syndromes, ultimately lowering the illness burden.

### Informed Consent Statement:

Before the study, informed consent was obtained from all subjects involved. Written informed consent was obtained from the patient’s guardian to publish this paper.

### Authors Contribution:

**RA:** Collected and sampled families, examined patient clinical data and conducted experiments.

**HA &RA:** Helped in analyzing whole exome sequencing data and assisted write-up.

**SMB, GH:** Designed the project, obtained financing, and edited the completed draft.
